# Laser ablation is superior to TACE in large-sized hepatocellular carcinoma: a pilot case–control study

**DOI:** 10.18632/oncotarget.24756

**Published:** 2018-04-03

**Authors:** Filomena Morisco, Silvia Camera, Maria Guarino, Raffaella Tortora, Valentina Cossiga, Anna Vitiello, Gabriella Cordone, Nicola Caporaso, Giovan Giuseppe Di Costanzo, Italian Liver Cancer (ITA.LI.CA) group

**Affiliations:** ^1^ Gastroenterology Unit, Department of Clinical Medicine and Surgery, University of Naples “Federico II”, Naples, Italy; ^2^ Hepatology Unit, “Cardarelli” Hospital, Naples, Italy

**Keywords:** large HCC, laser ablation, TACE

## Abstract

**Background:**

Limited therapies are available for large (≥40 mm) unresectable hepatocellular carcinoma (HCC). Currently, the standard treatment with transarterial chemoembolisation (TACE) is unsatisfactory with high recurrence rate and limited effect on survival. Laser Ablation (LA) has emerged as a relatively new technique characterized by high efficacy and good safety. This study is aimed to evaluate the efficacy of LA in comparison to TACE in patients with large HCC.

**Methods:**

Eighty-two patients with a single HCC nodule ≥40 mm (BCLC stage A or B) were enrolled in this case-control study. Forty-one patients were treated with LA and 41 patients were treated with TACE. Response to therapy was evaluated according to the mRECIST criteria. Survival was calculated with Kaplan-Meier from the time of cancer diagnosis to death with values censored at the date of the last follow-up.

**Results:**

Twenty-six (63.4%) and 8 (19.5%) patients had a complete response after LA and TACE, respectively (*p* < 0.001). Subsequently we stratified the HCCs in 3 categories according to the nodule size: 40–50 mm, 51–60 mm, and >60 mm. LA resulted superior to TACE especially in nodules ranging between 51 and 60 mm in diameter, with a complete response rate post-LA and post-TACE of 75% and 14.3%, respectively (*p* = 0.0133). The 36 months cumulative survival rate in patients treated with LA and TACE was 55.4% and 48.8%, respectively. The disease recurrence rates after LA and TACE were 19.5% and 75.0%, respectively.

**Conclusions:**

LA is a more effective therapeutic option than TACE in patients with solitary large HCC.

## INTRODUCTION

Hepatocellular carcinoma (HCC) is the fifth most frequent cancer among males and the ninth in women worldwide. In 2012, 782000 new HCC cases were registered [1]. The clinical and radiological presentations of HCC are extremely variable and a single HCC lesion with a diameter >40 mm is diagnosed in about 27% of cases *(ITA.LI.CA unpublished data*). In these patients the first line treatment is liver resection [2, 3]. Among patients unsuitable for surgery the treatment of solitary large HCC (>40 mm) is highly debated and its management represents a difficult challenge for clinicians. The standard treatment for these patients is transarterial chemoembolization (TACE) despite its unsatisfactory efficacy (complete response only in 25% of subjects [4]), with the occurrence of serious side effects in >10% of cases [5], and with an estimated 1-year recurrence rate of 59% [6]. Over the last two decades, local thermal ablative techniques have gained popularity and are considered the best treatment for unresectable early HCC with a size up to 30 mm [7, 8]. Percutaneous ablation has been attempted also in tumours larger than 30 mm, but these data are currently scarce. The most widely used techniques for treating large HCC are radiofrequency (RFA) and microwave (MWA) ablation with success rates of 83.4% and 86.7%, respectively [9]. Laser ablation (LA) is a less known and implemented in routine practice, despite the evidence of its non-inferiority compared to RFA in the treatment of early HCC [10]. Moreover, in a preliminary case-series study, LA with the multifiber technique achieved a complete response in 71% of nodules > 40 mm with mild side effects [11]. The aim of this case-control study was to evaluate the efficacy of LA in comparison to TACE in patients with unresectable solitary large HCC (≥ 40 mm). Our study was focused on this specific patients subgroup (solitary large HCC ≥ 40 mm) because traditional thermal ablation techniques (RFA and MWA) are considered less effective than TACE in obtaining a complete response [8–10].

## RESULTS

Baseline characteristics of the two groups of patients and HCC nodules are summarized in Table 1. No clinical and tumour findings were significantly different between the two groups, except for aetiology of underlying cirrhosis; HCV infection was more frequent in LA than in the TACE group (78% vs 53.7%; *p* = 0.036), while in the last group the alcoholic aetiology was more represented (19.5% vs 2.4%; *p* = 0.034).

**Table 1:** Baseline characteristics of the HCC population according to treatment options (LA vs TACE)

	LA group		TACE group		*p* value
	*n*	%	*n*	%	
**Male/Female**	29/12	(70.7/29.3)	29/12	(70.7/29.3)	1
**Age, years,** median (range)	72 (54–88)		72 (49–86)		0.603
**BMI**, median (range)	27.3 (17.2–36.6)		26.1 (17.7–32.0)		0.136
**Liver disease etiology**					
HCV infection	32	78.0	22	53.7	0.036
HBV infection	2	4.9	3	7.3	1
Alcohol	1	2.4	8	19.5	0.034
Others	6	14.7	8	19.5	1
**Comorbidities**					
none	13	31.7	16	39.0	0.508
metabolic	4	9.8	7	17.1	
cardiovascular	14	34.1	13	31.7	
metabolic + cardiovascular	9	22.0	5	12.2	
pulmonary	1	2.4	0	0.0	
**Child-Pugh class**					
A	34	82.9	37	90.2	0.519
B	7	17.1	4	9.8	
**BCLC stage**					
A	27	65.9	28	68.3	0.275
B	14	34.1	13	31.7	
**Tumour size, mm,** median (range)	46 (40–75)		47 (40–76)		0.16
**Nodule size >50 mm**	13 (31.7%)		14 (34.1%)		0.492

### Efficacy

LA approach resulted more effective than TACE in inducing a complete tumour ablation. Overall, 26 (63.4%) patients from the LA group and 8 (19.5%) from the TACE group showed a complete response to treatment (*p* < 0.001). At univariate analysis, baseline predictors of complete response were Child-Pugh class A and treatment modality with LA (Table 2).

**Table 2:** Univariate analysis of variables potentially related to complete tumour response

Variables	Complete response *n* = 34	Non responders *n* = 48	*p* value
**Male gender**	23 (67,6%)	35 (72.9%)	0.630
**Age > 70**	19 (55.9%)	30 (62.5%)	0.649
**Child-Pugh A**	33 (97.1%)	38 (79.2%)	**0.022**
**LA**	26 (76.5%)	15 (31.3%)	**<0.001**
**Nodule size <5 cm**	24 (70.6%)	28 (58.3%)	0.352

**Figure 1:**
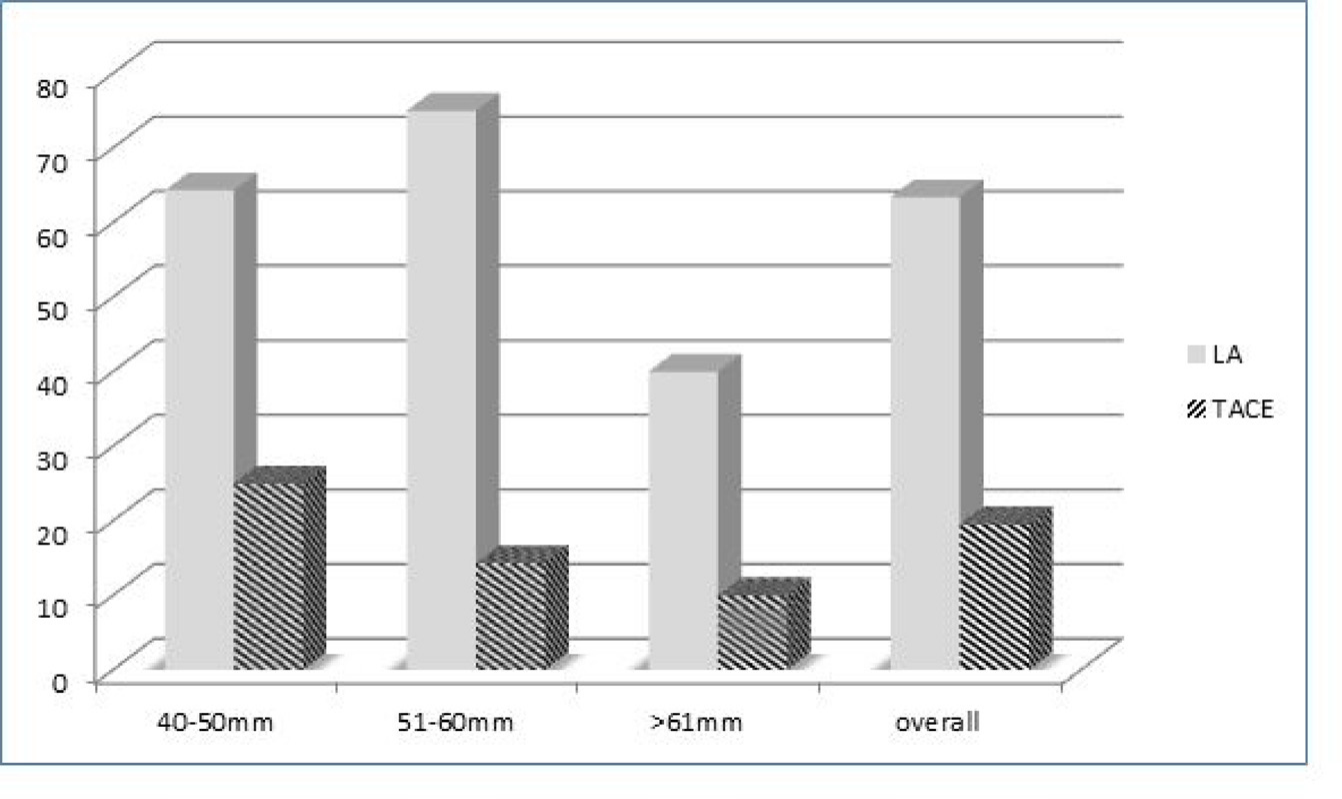
Complete response rates according to nodule size and treatment options (LA vs TACE)

Furthermore, HCC were stratified into 3 categories according to the nodule size: 40-50 mm, 51-60 mm, and >60 mm. The complete response rate according to nodule size is reported in Figure 1. LA resulted more effectively than TACE in all the categories, especially in nodules with the diameter ranging between 51 and 60 mm, with complete response rates after LA and TACE of 75% and 14.3%, respectively (*p* = 0.013).

### Disease recurrence rate

During a mean ± SD follow-up period of 37.4 ± 20.7 months (LA 37.8 ± 20.9–TACE 37.0 ± 20.7) the disease recurrence was observed in 5/26 LA-successfully treated patients (19.5%) and in 6/8 TACE-successfully treated patients (75.0%) (*p* < 0.0001). The mean times to recurrence was 42.2 months (95% CI, 34.3-50.1) and 26.8 months (95% CI, 18.6-34.9) in LA and TACE group, respectively (*p* = 0.004). Mean disease-free survival period were 31.5 months (95%CI, 24.1-38.8) among LA group patients and 14.2 months (95% CI, 9.7–18.7) among TACE group patients (*p* < 0.0001; HR 0.64).

At univariate analysis, time to recurrence was only influenced by treatment modality (LA vs TACE) and MELD value (Table 3).

**Table 3:** Univariate analysis of variables potentially related to HCC time to recurrence

	Mean TTR *(months)*	95%CI	*p*
**LA**	42.19 ± 4.01	34.326–50.069	**0.004**
**TACE**	26.77 ± 4.17	18.590–34.951	
**Child-Pugh A**	33.664 ± 3.270	27.254–40.073	0.592
**B**	39.400 ± 7.995	23.729–55.071	
**BCLC A**	30.938 ± 3.270	22.900–38.977	0.405
**B**	38.137 ± 4.504	29.309–46.964	
**MELD >10**	24.748 ± 4.946	15.053**–**34.443	**0.018**
**≤ 10**	39.357 ± 3.716	32.074–46.640	

TTR: Time to recurrence.

### Survival analysis

**Figure 2:**
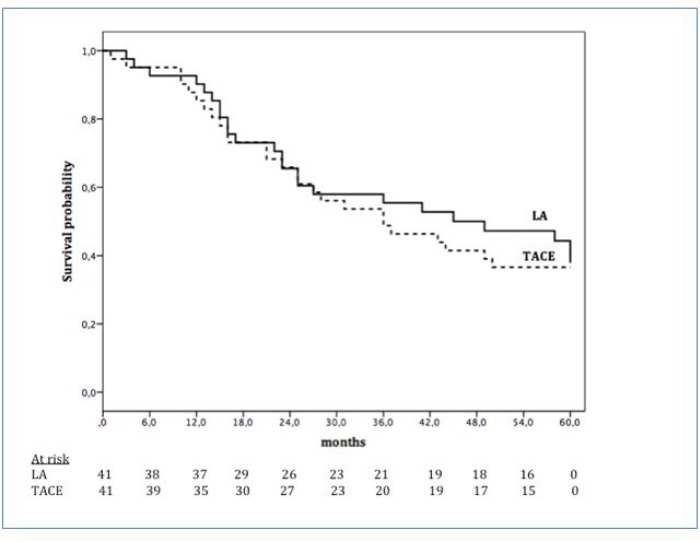
Overall survival according to treatment options: LA (continuous line) and TACE (dotted line)

The mean OS was 38.3 months (95% CI, 33.8-42.9). Among the LA patients, it was 39.7 months (95% CI, 33.1-46.4) while 37.0 months (95% CI, 30.7-43.3) for TACE group (*p* = 0.725) (Figure 2). Overall survival probability rates at 1-year, 2-years, and 3-years were 90.2%, 65.5%, and 55.4% in LA group and 85.4%, 65.9%, and 48.8% in TACE group (Figure 2).

During the study period 50 patients died; 24 patients in LA group and 26 in TACE group. The cause of death was unknown in 6 patients of LA group and in 1 patient of TACE group. HCC progression was observed in 53% (12 patients) and 64% (16 patients) and liver failure in 29% (6 patients) and 36% (9 patients) of LA and TACE group, respectively.

## DISCUSSION

Over the past 20 years laser ablation technique has been developed and improved for the treatment of HCC [10]. Cohort series, retrospective studies and a non-inferiority randomized controlled trials demonstrated that this technique is effective in treating HCC and is non-inferior to the widely used RFA [12–15] in nodules <30 mm. On the other hand, information for nodules >40 mm derives from a single preliminary report using the multifiber technique achieving a complete response in 71% of nodules [11].

Currently, the standard treatment for patients with solitary large HCC (≥40 mm) is TACE, despite its suboptimal rate of complete ablation (about 25%) in relation to the nodule size [4] and with serious side effects in > 10% of cases [5].

Alternative therapeutic procedures have been proposed such as transarterial radioembolization (TARE), a selective intra-arterial liver injection using yttrium-90-loaded microspheres. This type of treatment is considered a good choice in large tumours (up to 10 cm) especially with multiple satellite nodules [16]. The complete response rate after TARE is 40-50%, although the rate of efficacy derives almost exclusively by a large and heterogeneous cohort of subjects with both solitary or multifocal large HCC [16].

Monopolar RFA is the most widely used technique to perform local ablation. Its efficacy is reduced when it is used for treating nodules larger than 30 mm. In these cases, multiple overlapping ablations or switching the RFA mode using three separate needles have been used [17, 18]. Multipolar RFA with three electrodes is less investigated, but seems promising to increase the volume of the ablated area [19]. In recent years, MWA is emerging as an alternative to RFA for thermal destruction of HCC because it induces higher intratumoral temperature in a very short time. In two recent meta-analyses, this technique seems to reduce the local recurrence rate in large nodules (≥50 mm) as compared to RFA [20, 21].

A clear state-of-the-art treatment of large HCC remains an unmet clinical need and more effective therapies are required.

To better explore the efficacy of LA for treatment of large solitary unresectable HCC, we conducted this case-control study comparing LA with TACE, that is considered the current standard of care [22].

Our study indicates that multifiber LA approach is more effective than TACE in treating solitary large (≥40 mm) unresectable HCC, by obtaining a complete tumour ablation (achieved in about 2/3 of patients, 63.4%) and by reducing the recurrence rate (19.5% vs 75.0%, both with a statistically significant difference).

Overall LA was superior to TACE in all the nodule-size categories, but especially in nodules with diameters ranging between 51 and 60 mm, with a complete response rate of 75% in comparison to complete response of 14.3% observed after TACE procedure. Differently to TACE, LA shows efficacy in relation to nodule size [4]. Its effectiveness seems to be regardless of the nodule size, at least for nodules sizing less than 60 mm. For nodules with diameter >60 mm a reduction of the complete response rate was observed, although the LA remains more efficacious than TACE. In addition to a more efficient ablation, LA is able to reduce the rate of recurrence.

The local recurrence rate after TACE widely varies from 30 to 70 % across published studies [4, 23, 24].

In our experience in patients treated with LA the recurrence rate was 19.5% compared to 75.0% of TACE-treated patients. Thus, LA is able to halve the disease recurrence rate during a follow-up period of 37 months. The principal reason for the better outcome is probably due to a higher effectiveness in term of local tumour control with larger ablative margins and the elimination of peripheral satellite nodules or occult foci of cancer cells [25, 26].

The survival rates of our patients at 1-year, 2-years, and 3-years were 90.2%, 65.5%, and 55.4% in LA group and 85.4%, 65.4%, and 48.8% in TACE group, without any statistically significant differences between the two procedures. Although there is no clear explanation for the discrepancy between primary effectiveness rate (complete lesion ablation) and the long term outcome (survival), the literature suggests that the two outcomes do not necessarily overlap [27]. On the other hand, a large series of studies reports that survival depends not only by the efficacy of treatment but also by several factors like age, comorbidities, stage of chronic liver disease, nodule size, and efficacy of subsequent treatment of HCC in patients with residual lesions. Our series of patients are well-matched for these factors, although some small differences not statistically significant, could potentially explain the survival rates in the 2 groups of patients.

Additionally, very poor information is available in the literature regarding long-term survival rates after treatment of patients with solitary large HCC. In a series of 64 patients with large lesion treated with MWA vs < TACE, the survival rates after 12 and 18 months of follow-up were 78.2% and 68.4% in the MWA group and 52.4% and 28.8% in the TACE group, respectively [28]. Contrarily, our study is the first reporting the efficacy of LA in solitary large nodules with a long-term follow-up period (3 years).

Our study has some limitations that we now briefly discuss. Firstly, our work uses an historical control group and therefore it may suffer from unintended biases specifically related to the retrospective design of the database. Secondly, the small sample size of the two analyzed groups could be a limiting factor as statistical tests normally require a larger sample size to ensure a representative distribution of the population and generalized extrapolation. Nonetheless, it should be underlined that this particular HCC “stage” specifically treated with this approach is not common. Finally, the comparison between a single center cohort with a multicenter cohort could represent another bias as per definition single center cohorts concentrate expertise while multicenter cohorts include different kind of centers, despite the fact that the ITALICA group is composed by Italian expert centers in HCC treatment.

The indisputable advantage of this study is that it benefit from a single-center group of LA patients with a the long-term operator experienced in performing LA. To the best of our knowledge, this study represents the first and largest cohort with single large HCCs, exclusively investigated for treatment with LA with a long term outcome. This is of particular relevance given the limited therapies available for large unresectable HCC with a curative intent.

In conclusion, the results of this study suggest that LA may be a valuable alternative to TACE in the treatment of patients with large HCC in which surgical resection is unsuitable or inappropriate. LA was superior to TACE especially in nodules with a diameter ranging between 40 and 60 mm. Nevertheless, larger well-designed randomized controlled trials are needed to confirm our results.

## MATERIALS AND METHODS

### Patients

This is a retrospective case-control study approved by the local institutional review board. Inclusion criteria were: i) unresectable HCC (due to nodule location, presence of portal hypertension, age >75 years, or comorbidities) or refusal of surgery; (ii) solitary HCC ≥40 mm; (iii) BCLC stage A or B; (iv) Child–Pugh class A or B cirrhosis; (v) platelet count > 40 000/μL and INR <2.0; and (vi) no history of previous HCC treatment. Exclusion criteria included: (i) history of encephalopathy or refractory ascites; (ii) vascular invasion or extrahepatic metastasis; (iii) severe comorbidities reducing life expectancy; (iv) BCLC stage C or D; (v) Child–Pugh class C cirrhosis.

Between January 2009 and December 2012, a total of 432 naïve HCC cirrhotic patients were consecutively observed at the Liver Unit of Cardarelli Hospital, Naples, Italy. Among them, 41 consecutive cirrhotic patients with a single HCC nodule ≥ 4.0 mm in diameter met the entry criteria and were enrolled into the study. All 41 patients were treated with LA treatment.

The control group was obtained from the Italian Liver Cancer (ITA.LI.CA.) database [29] and was represented by 41 patients who met the same inclusion and exclusion criteria undergoing a TACE treatment within the same time-frame.

The diagnosis of HCC was based on the European Association for the study of the Liver (EASL) guidelines for HCC management [22]. The patients were classified according to Barcelona Clinic Liver Cancer (BCLC) Staging System [30]. The size and number of the HCC lesions and their location in the liver were established by ultrasound and contrast enhanced Computed Tomography (CT) or Magnetic Resonance Imaging (MRI). In particular, for both diagnostic work-up and follow-up, CT scan and MRI were considered equally given the large size of the nodules [31]. Portal hypertension was defined according to the EASL guidelines.

### Treatment procedures

The term laser ablation refers to the thermal destruction of tissue by conversion of absorbed light (usually infrared) into heat. Infrared energy penetrates tissue directly for a distance of 12–15 mm, although heat is conducted beyond this range creating a larger ablative zone [31]. Optical penetration has been shown to be increased in malignant tissue compared to normal parenchyma. The details of LA procedure have been reported elsewhere [27, 32, 33].

In the present study LA was performed with the multifiber technique. In cases of nodules up to 50 mm four fibers arranged in a square configuration were employed; for larger nodules eight fibers positioned in a two-square configuration were used. Twenty-five and 7 patients received respectively two and three LA treatment sessions. Patients with residual cancer subsequently received combined treatments (thermal ablation plus TACE) and in case of no response to this combined modality sorafenib was used.

Conventional TACE was performed by injection of epirubicin, lipiodol and an embolizing agent after selective catheterization of the hepatic arteries feeding the lesion, according to the standardized protocol (all of the enrolled patients received at least 2 TACE treatment sessions) [34].

### Outcomes and assessments

The main outcome was the complete response rate to treatment. Response to therapy was evaluated with contrast enhanced CT or MRI twelve weeks after the last TACE or LA session according to the modified Response Evaluation Criteria in Solid Tumours (mRECIST) [33]. Complete response after LA was defined as the disappearance of any intratumoral arterial enhancement in target lesions. The response after TACE was defined complete when a homogeneous uptake of lipiodol and a complete absence of intratumoral enhancement was observed in the target lesions at CT scan or when MRI showed absence of any enhancement within the nodules.

Secondary outcomes were the evaluation of local tumour progression (LTP) and overall survival (OS). All patients underwent follow-up investigations, including α-fetoprotein measurement and ultrasonography assessment every 3 months, CT or MRI every 6 months, and in any suspected case of tumoural recurrence. LTP was defined as reappearance of arterial enhancement on CT or MRI either within a treated tumour or near its margins. OS was calculated from the time of cancer diagnosis to death with values censored at the date of the last follow-up. For the TACE group, details on the ITA.LI.CA data base management have been already reported [35].

### Statistical analysis

Categorical variables were summarized as numbers and percentages; continuous variables were presented as median, range, standard deviation (SD) and 95% confidence intervals (95% CI). Categorical data were compared using a Chi-square test and continuous variables by Mann-Whitney test. The two-sided probability value <0.05 was considered statistically significant. Kaplan-Meier method was used to estimate overall survival. Survival was calculated from the date of diagnosis to the date of death. Patients who were alive at the time of the analysis were censored at the last follow-up time. Differences in survival times between groups were assessed by the log-rank test.

Univariate analysis to search for factors associated to complete ablation, recurrence-free survival and OS was performed evaluating Child-Pugh class (A vs B), MELD score (<10 vs ≥10), BCLC class (A vs B), and treatment (LA vs TACE). All of the analyses were performed with software package SPSS for Mac (Rel SPSS 21.0; IBM corporation, 2012).

### Members of the ITA.LI.CA. group

Dipartimento di Scienze Mediche e Chirurgiche, Alma Mater Studiorum – Università di Bologna: Zoli M, Garuti F, Neri A, Piscaglia F, Lenzi B, Valente M, Trevisani F, Bolondi L, Biselli M, Caraceni P, Cucchetti A, Domenicali M, Gramenzi A, Magalotti D, Serra C, Venerandi L; Dipartimento di Malattie Apparato Digerente e Medicina Interna, Azienda ospedaliero universitaria di Bologna, Unità Operativa di Radiologia: Cappelli A, Golfieri R, Mosconi C, Renzulli M; Dipartimento di Medicina Interna, Unità di Gastroenterologia, Policinico San Martino, Università di Genova: Giannini EG, Brunacci M, Moscatelli A, Pellegatta G, Savarino V; Unità Operativa di Gastroenterologia, Ospedale Belcolle, Viterbo: Caturelli E, Roselli P, Lauria V, Pelecca G; Unità Operativa di Medicina Protetta, Ospedale Belcolle, Viterbo: Dell’Isola S, Ialungo AM, Rastrelli E; Dipartimento Biomedico di Medicina Interna e Specialistica, Unità di Gastroenterologia, Università di Palermo: Cabibbo G, Cammà C, Attardo S, Rossi M, Cavani G; Dipartimento Biomedico di Medicina Interna e Specialistica, Unità di Medicina Interna 2, Azienda Ospedaliera Ospedali Riuniti Villa Sofia-Cervello, Palermo: Virdone R, Affronti A; Dipartimento di Medicina Clinica e Chirurgia-Università Federico II, Napoli: Nardone G; Ospedale Regionale di Bolzano, Unità di Gastroenterologia, Bolzano: Felder M, Mega A; Unità Operativa di Chirurgia, Policlinico S. Marco, Zingonia: Ciccarese F, Del Poggio P, Olmi S; Dipartimento di Medicina Interna; Ospedale per gli Infermi di Faenza, Faenza: Foschi FG, Bevilacqua V, Dall’Aglio AC, Ercolani G, Fiorini E, Casadei Gardini A, Lanzi A, Mirici Cappa F; Unità Operativa Gastroenterologia e Malattie del Ricambio, Azienda Ospedaliero-Universitaria Pisana, Pisa: Sacco R, Mismas V; Clinica di Gastroenterologia, Università Politecnica delle Marche, Ancona:Svegliati Barone G, Schiadà L; Dipartimento di Scienze Chirurgiche e Gastroenterologiche, Università di Padova: Farinati F, Gazzola A, Murer F, Pozzan C, Vanin V; Unità di Medicina Interna e Gastroenterologia, Complesso Integrato Columbus, Università Cattolica di Roma, Roma: Rapaccini GL, de Matthaeis N; Unità di Medicina Interna e Gastroenterologia, Policlinico Gemelli, Università Cattolica di Roma, Roma: Gasbarrini A, Rinninella E; Unità di Malattie Infettive ed Epatologia, Azienda Ospedaliero-Universitaria di Parma: Olivani A, Missale G, Biasini E; Unità Operativa di Medicina, Azienda Ospedaliera Bolognini, Seriate, Italia: Di Marco M, Balsamo C, Vavassori E; Unità di Gastroenterologia, Ospedale Sacro Cuore Don Calabria, Negrar: Masotto A, Marchetti F, Valerio M; Medicina Interna ed Epatologia, Dipartimento di Medicina Sperimentale e Clinica, Firenze: Marra F, Aburas S, Campani C, Dragoni G; Dipartimento di Medicina, Medicina Interna ed Epatologia, Ospedale Fatebenefratelli, Milano: Borzio F; Dipartimento di Medicina Molecolare Università di Padova: Benvegnù L; Dipartimento di Scienze Mediche Chirurgiche, Unità di Gastroenterologia, Alma Mater Studiorum - Università di Bologna, Bologna: Festi D, Giovanni Marasco, Federico Ravaioli.
